# Cross Attention Transformers for Multi-modal Unsupervised Whole-Body PET Anomaly Detection

**DOI:** 10.1007/978-3-031-18576-2_2

**Published:** 2022-10-08

**Authors:** Ashay Patel, Petru-Daniel Tudosiu, Walter Hugo Lopez Pinaya, Gary Cook, Vicky Goh, Sebastien Ourselin, M. Jorge Cardoso

**Affiliations:** 1 King’s College London, London, WC2R 2LS, United Kingdom

**Keywords:** Transformers, Unsupervised Anomaly Detection, Cross-Attention, Multi-modal, Vector Quantized Variational Autoencoder, Whole-Body, Kernel Density Estimation

## Abstract

Cancers can have highly heterogeneous uptake patterns best visualised in positron emission tomography. These patterns are essential to detect, diagnose, stage and predict the evolution of cancer. Due to this heterogeneity, a general-purpose cancer detection model can be built using unsupervised learning anomaly detection models; these models learn a healthy representation of tissue and detect cancer by predicting deviations from healthy appearances. This task alone requires models capable of accurately learning long-range interactions between organs, imaging patterns, and other abstract features with high levels of expressivity. Such characteristics are suitably satisfied by transformers, and have been shown to generate state-of-the-art results in unsupervised anomaly detection by training on healthy data. This work expands upon such approaches by introducing multi-modal conditioning of the transformer via cross-attention, i.e. supplying anatomical reference information from paired CT images to aid the PET anomaly detection task. Using 83 whole-body PET/CT samples containing various cancer types, we show that our anomaly detection method is robust and capable of achieving accurate cancer localisation results even in cases where healthy training data is unavailable. Furthermore, the proposed model uncertainty, in conjunction with a kernel density estimation approach, is shown to provide a statistically robust alternative to residual-based anomaly maps. Overall, a superior performance is demonstrated against leading alternatives, drawing attention to the potential of these approaches.

## 1 Introduction

Positron Emission Tomography (PET) promises one of the highest detection rates for cancer amongst imaging modalities [14]). Through enabling the visualization of metabolic activity, the efficacy of PET is brought down to the high metabolic rates of cancer cells [1]. By detecting changes on a cellular level, PET is ideal for detecting new and recurrent cancers [13]. In most clinical applications, however, PET is coupled with CT or MRI data to allow the incorporation of structural information with the results presented from PET imaging.

Cancer detection and segmentation present a wide range of clinically relevant tasks from staging, treatment planning, and surgical or therapy intervention planning. Although effective, PET imaging sensitivities can range as much as 35% depending on the cancer type and radiologist [17]. This can be of further issue in the case of metastatic cancer where dissemination can easily be overlooked in small, superficial lesions [20]. Considering these shortfalls, there is significant motivation for developing accurate automated detection methods.

Unsupervised methods have become an increasingly prominent field in recent years for automatic anomaly detection by eliminating the necessity of acquiring accurately labelled data [5, 2]. These methods mainly rely on creating generative models trained on healthy data. Then during inference, anomalies are defined as deviations from the defined model of normality. This approach eliminates the requirement of labelled training data and generalises to unseen pathologies. However, its efficacy is often limited by the requirement of uncontaminated data with minimal anomalies present during training. The current state-of-the-art models for the unsupervised generative approach are held by the variational autoencoder (VAE) and its variants. In Baur et al. [2] VAE approach, the healthy data manifold is obtained by constraining the latent space to conform to that of a given distribution. The reconstruction error is then used to localise anomalies during inference. This approach, however, has limitations: from low fidelity reconstructions to the lack of resilience to reconstructing anomalous data.

To overcome some of these issues, an approach for unsupervised anomaly detection was presented utilising autoregressive models coupled with vector-quantised variational autoencoder (VQ-VAE) [18, 15].

Transformers, who are currently state-of-the-art networks in the language modelling domain [25, 22], use attention mechanisms to learn contextual dependencies regardless of location, allowing the model to learn long-distance relationships to capture the sequential nature of sequences. This general approach can be generalised to any sequential data, and many breakthroughs have seen the application of transformers in computer vision tasks [5, 6, 12, 26]. Although having showcased state-of-the-art performance in unsupervised anomaly detection tasks for medical imaging data [21], these methods still rely on healthy data for model training. To the best of our knowledge, no prior research exists using unsupervised methods to accurately localise abnormalities while using training data containing anomalies. This task is important as it is often difficult or unethical to obtain healthy datasets of certain medical imaging modalities as some images are only acquired with prior suspicion of disease.

To address these problems, we propose a method for unsupervised anomaly detection and segmentation using multi-modal imaging via transformers with cross attention. This method is able to detect anomalies even when trained on anomalous data by leveraging the heterogeneity of metastatic cancer and anatomical information from CT. Furthermore utilising the generative aspect of the transformer model we propose and evaluate a kernel density estimation approach for generating a more robust alternative to residual based anomaly maps.

## 2 Background

The principal components behind the proposed whole-body anomaly detection model rely on using transformer models and auto-encoders to learn the probability density function of 3D whole-body PET scans. Although all training data contain anomalies, the spread of metastatic cancer and spatial distribution of anomalies across samples will result in such anomalies being unlikely, thus appearing at the likelihood tail-end of the learnt distribution. In order to use transformer models, images need to be expressed as a sequence of values, ideally categorical. As it is not computationally feasible to do this using voxel values, a compact quantized (discrete) latent space is used as input for the transformer via a VQ-GAN model [18, 10] (a VQ-VAE with an adversarial component).

### 2.1 VG-GAN

The original VQ-VAE model [18] is an autoencoder that learns discrete latent representations of images. The model comprises of three principal modules: the encoder that maps a given sample *x* ∈ ℝ^*H*×*W*×*D*^ onto a latent embedding space $\hat{z}\in {\mathbb{R}}^{h\times w\times d\times n_{z}}$ where *n*_*z*_ is the size of each latent vector. Each latent vector is quantized using an element-wise quantization of which each code ${\hat{z}}_{ijl}\in {\mathbb{R}}^{n_{z}}$ is mapped to its nearest vector *e*_*k*_, *k* ∈ 1, …*K*, where *K* is the vocabulary size of a codebook learnt jointly with model parameters. The final portion of the network is the decoder, which reconstructs the original observation from the quantized latent space. The discrete latent space representation is thus a sequence of indexes *k* for each code from the codebook. As autoencoders often have limited fidelity reconstructions [9], as proposed in [10], an adversarial component is added to the VQ-VAE network to form a VQ-GAN. Further formulations and architecture details can be found in appendix B.

### 2.2 Transformer

Once a VQ-GAN model is trained on the entire training set containing anomalous data, the following stage is to learn the probability density function of the sequence of latent representations in an autoregressive manner. Transformer models rely on attention mechanisms to capture the relationship between inputs regardless of the distance or positioning relative to each other. Within each transformer layer, a self-attention mechanism is used to map intermediate representations with three vectors: query, key and value (see appendix C for detailed formulation). This process, however, relies on the inner product between elements and as such, network sizing scales quadratically with sequence length. Given this limitation, achieving full attention with large medical data, even after the VQ-GAN encoding, comes at too high a computational cost. To circumvent this issue, many efficient transformer approximations have been proposed [24, 7]. In this study, a Performer model is used; the Performer makes use of the FAVOR+ algorithm [7] which proposes a linear generalized attention that offers a scalable estimate of the attention mechanism. In using such a model, we can apply transformer-like models to much longer sequence lengths associated with whole-body data. In order to learn the probability density function of whole-body data, the discretised latent space *z*_*q*_ must take the form of a 1D sequence *s* using some arbitrary ordering. We then train the transformer model to maximise the training data’s log-likelihood in an autoregressive manner. In doing so, the transformer learns the distribution of codebook indices for a given position *i* with respect to all previous inputs *p*(*s*_*i*_) = *p*(*s*_*i*_ | *s*_*<i*_).

## 3 Method

### 3.1 Anomaly Detection

To perform the baseline anomaly detection model on unseen data, first, we obtain the discrete latent representation of a test image using the VQ-GAN model. Next, the latent representation *z*_*q*_ is reshaped using a 3D raster scan into a 1D sequence *s* where the trained Performer model is used to obtain likelihoods for each latent variable. These likelihoods represent the probability of each token appearing at a given position in the sequence *p*(*s*_*i*_) = *p*(*s*_*i*_ | *s*_*<i*_), highlighting those of low probability of appearing in healthy data. Then tokens with likelihoods below an arbitrary threshold are selected to generate a binary resampling mask to indicate abnormal latent variables *p*(*s*_*i*_) *< t* (where *t* is a threshold determined empirically using a validation dataset; *t* = 0.01 was found to be optimal). Using the resampling mask, the latent variables are “healed” by resampling from the transformer and replacing them in the sequence. This approach replaces anomalous latent variables with those that are more likely to belong to a healthy distribution. Using the “healed” latent space, the VQ-GAN model reconstructs the original image *x* as a healed reconstruction *x*_*r*_. Finally, a voxel-wise residual map can be calculated as *x*–*x*_*r*_ with final segmentations calculated by thresholding the residual values. As areas of interest in PET occur as elevated uptake, residual maps are filtered to only highlight positive residuals.

### 3.2 CT Conditioning

**Fig. 1:**
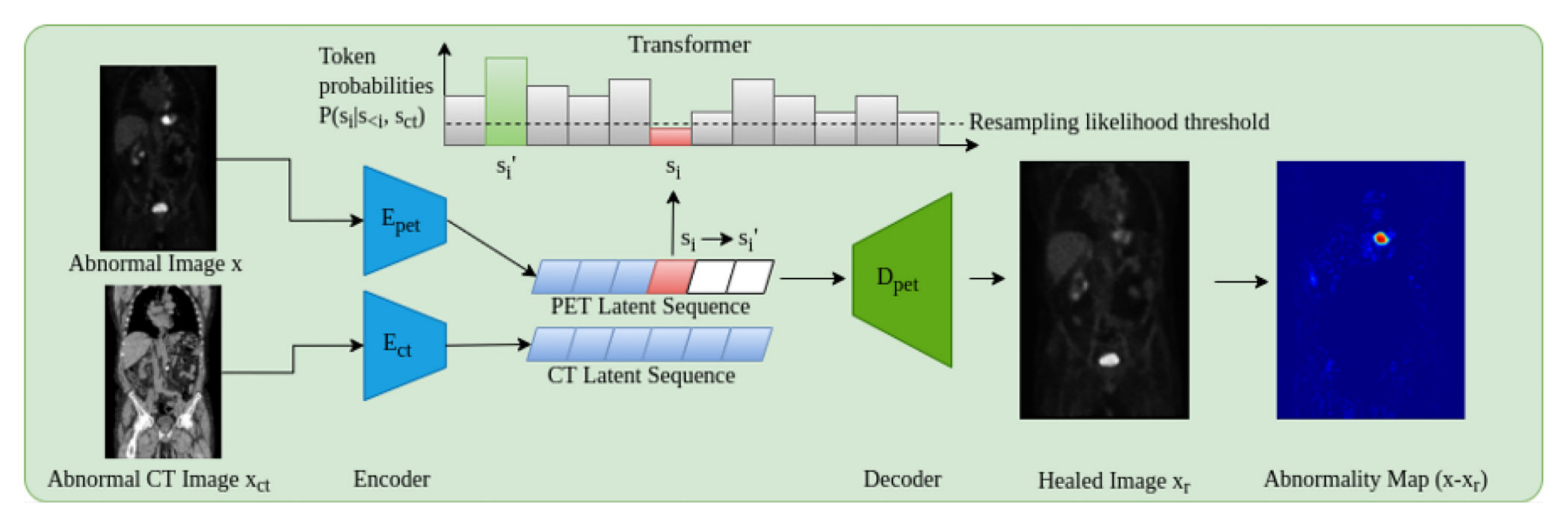
Anomaly Detection Pipeline - PET image *x* is encoded along with CT image *x*_*ct*_. Tokens from the encoded PET image are then sampled from the transformer by obtaining their likelihood with respect to prior tokens in the sequence and all CT tokens. Tokens below a given threshold are resampled from a multinomial distribution, derived from likelihood outputs from the transformer for all tokens at a given position in the sequence, giving a "healed" latent space which is decoded to give *x*_*r*_.

There are often times when more information can be useful for inference. This can be in the imaging domain through multiple resolutions [4], or multiple modalities/spectrums [16]. It is for these tasks where cross-attention can prove beneficial. From a clinical point of view, whole-body PET scans are acquired in conjunction with MRI or CT data to provide an anatomical reference as structural information. Additionally, it can be observed that areas of high uptake are not always associated with anomalies. For example, areas of high metabolic activity like the heart, in addition to areas where radiotracer may collect like the kidney and bladder can show high uptake patterns. Sometimes these areas are not obvious from PET alone, and as such, the anatomical reference provided from CT data is beneficial. This leads to the main contribution of the work, namely anomaly detection incorporating CT data. This process works by generating a separate VQ-GAN model to reconstruct the PET-registered CT data. Then, both CT and PET data are encoded and ordered into a 1D sequence using the same rasterisation process, such that CT and PET latent tokens are spatially aligned. The transformer network is then adapted to include cross-attention layers [11] that feed in the embedded CT sequence after each self-attention layer. At each point in the PET sequence, the network has a full view of the CT data helping as a structural reference. In doing so, the problem of determining the codebook index at a given position *i* becomes *p*(*s*_*i*_) = *p*(*s*_*i*_ | *s*_*<i*_, *c*) where *c* is the CT latent sequence (detailed formulation can be found in appendix C). This approach, as visualised in Fig. 1 adds robustness to the anomaly detection framework by providing meaningful context in areas of greater variability in uptake that can be explained by the anatomical information within CT.

### 3.3 Kernel Density Estimation

A drawback of the baseline anomaly detection method is that the residual image uses an arbitrary threshold to generate a segmentation map. The resulting segmentation can often be noisy due to discrepancies between the reconstructed image and the original, for example, between borders of high-intensity. Additionally, anomalies can occur at different intensities, meaning a blanket threshold is not appropriate. A possible solution to this is to implement Z-score anomaly maps as used in similar anomaly detection work [3]. For this work, this can be achieved by introducing variability within the model. However, certain uptake patterns can be related to base metabolic rate, in addition to procedure-related variations such as injected tracer amount and time since injection. As such, the optimality of the Z-score’s Gaussian-error assumption should be questioned and likely relaxed. Empirical evidence obtained by exploring the data and by sampling from the transformer itself highlights that the error is indeed non-Gaussian even in healthy regions, for example the heart; bi-modal (even multi-modal) error distributions are observed. To remedy this, we propose to use a non-parametric approach using kernel density estimation (KDE) [19]. To do this, we introduce model uncertainty by using a dropout layer in the VQ-GAN decoder. Additionally, we obtain variability through replacing unlikely tokens with ones drawn from a multinomial distribution, derived from the likelihoods output from the transformer for each token at a given position in the sequence. By sampling multiple times, we generate multiple "healed" latent representations for a single image, which are then decoded multiple times with dropout to generate multiple "healed" reconstructions of a sample. At which point a KDE is fit at each voxel position to generate an estimate of the probability density function *f*. Letting (*x*_1_, …, *x*_*n*_) be the intensity for a voxel position across reconstructions, we can generate an estimation for the shape of the density function *f* for voxel *x* as:

(1) $${\hat{f}}_{h}(x)=\frac{1}{nh}\overset{n}{\underset{i=1}{\sum }}K\;(\frac{x-x_{i}}{h})$$

where *K* is a gaussian kernel, and *h* is a smoothing bandwidth calculated as

(2) $$h={\left(\frac{4{\hat{\sigma }}^{5}}{3n}\right)}^{1/5}$$

with $\hat{\sigma }$ representing the standard deviation at a given voxel position across *n* reconstructions. We can then score voxels from that estimated density function at the intensity of the real image, at the voxel level, to generate a log-likelihood for that intensity, generating the anomaly map. To address areas of low variance across reconstructions, we implemented a minimum bandwidth of 0.05 (determined empirically using a validation dataset).

### 3.4 Clinically Consistent Segmentations for PET

For whole-body PET, the contours of an anomaly can be hard to define. The clinical standard in the UK defines boundaries of an anomaly as connecting voxels with intensities above 40% of the maximum intensity of a specific anomaly. To conform to this standard, we apply a final post-processing step of growing all initial segmentations to satisfy this criteria.

## 4 Results

The proposed models were trained on 60 images, with model and anomaly detection hyperparameter tuning carried out on 11 validation samples using the best DICE scores. To assess our method’s performance, we use 12 hold-out paired whole-body PET/CT images with varying cancers. We measure our models’ performance using the best achievable DICE score, which serves as a theoretical upper-bound to the models segmentation performance. We obtained the scores using a greedy search approach for residual/density score thresholds. In addition, we calculate the area under the precision-recall curve (AUPRC), as a suitable measure for segmentation performance under class imbalance. We also compare our results to that of a VAE model proposed in [2]. Finally, we performed an ablation study of the proposed methods to demonstrate their added contribution along with paired t-tests to showcase the statistical significance of improvements.

### Ablation study

**Fig. 2:**
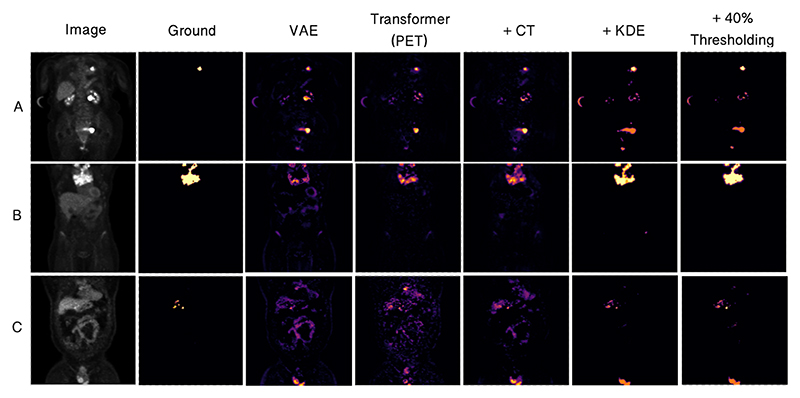
Columns from left to right display (1st) the input image; (2nd) the gold standard truth segmentation; (3rd) the abnormality map as the residual for the VAE, (4th) Transformer, and (5th) CT conditioned methods; (6th) the abnormality map as a KDE, (7th) and after thresholding at 40% of each abnormal region maximum value. Results are provided for four randomly chosen subjects (A,B,C).

We observe a considerable improvement (*P* = .001) in anomaly detection performance by implementing CT conditioning in comparison to the 3D GAN variant approach of [21]. This result confirms our initial thoughts on the use case of anatomical context in the case of whole-body PET. Given the variability of healthy radiotracer uptake patterns, it is expected that beyond common areas like the bladder, further context is required to identify uptake as physiological or pathological. By incorporating model uncertainty to generate KDE maps, we see a further improvement in the overall DICE score, and even greater increase in AUPRC from 0.344 to 0.501 against the CT conditioned model (*P <* .001). This behaviour can be explained by the increased variability around heterogeneous areas of healthy uptake, attributing to a decrease in false positives. The main advantage of this approach, as visualised in Fig. 2 is the increase in precision. By discarding the assumption of Gaussian uptake distributions, the model can better differentiate patterns of physiological uptake from pathological whilst still being sensitive to subtle anomalies, as seen in sample C in Fig. 2.

### Comparison to state-of-the-art

From Table 1, we can see a statistically-significant improvement (*P* = .001) presented via the VQ-GAN + transformer approach using only PET data in relation to the VAE. This result is expected as demonstrated in prior research [21]. However, this divergence is also attributed to the presence of anomalies during training. It can be observed from sample B in Fig. 2, that the autoencoder method attempts to reconstruct large anomalies. Comparing the method proposed by [21] to our best model comprising of CT conditioning and KDE anomaly maps, our approach generates an improvement in DICE score from 0.424 to 0.505 (*P <* .001) with a considerable increase in AUPRC from 0.301 to 0.501 (*P <* .001). Finally, through clinically accurate segmentations by growing segmented regions, we see a large increase in the best possible DICE score, but a reduction in AUPRC brought about by the expansion of false-positive regions. From the results, there is clear evidence and motivation for the use of multi-modal conditioning for whole-body PET anomaly detection. In general from the qualitative results we can see detection results are high even from the PET only transformer approach however the incorporation of CT helps to improve precision through improved knowledge of the anatomical regions in the scan. Additionally the use of KDE based anomaly maps showcase a significant improvement on residual based maps. However, there are still areas for improvement beyond the current scope. We see varying cases of false positives across samples, showing ongoing difficulties differentiating physiological uptake from pathological. The reasons may be due to patient factors, i.e. general health, or more procedure-based factors, including radiotracer dosage and time since injection. Naturally, one solution would be to provide more training data; however, an alternative is to provide further conditioning related to the patient and procedure.

## 5 Conclusion

Detection and segmentation of anomalous regions, particularly for cancer patients, is essential for staging, treatment and intervention planning. In this study, we propose a novel pipeline for a transformer-based anomaly detection approach using multi-modal conditioning and kernel density estimation via model uncertainty. The model achieves statistically-significant improvements in Dice and AUPRC, representing a new state-of-the-art compared to competing methods. Additionally, we show the impact of this approach when faced only with training data containing anomalies, showing greater robustness than autoencoder only approaches. We hope that this work will inspire further investigation into anomaly detection with conditioned transformers using multi-modal medical imaging, and further exploration into the development of these methods.

## Supplementary Material

Appendix

## Figures and Tables

**Table 1:** Anomaly detection results on whole-body PET data. The performance is measured with best achievable DICE-score (⌈*DICE*⌉) and AUPRC on the test set.

Method	⌈*DICE*⌉	AUPRC
VAE (Dense)[2]	0.359	0.282
VQ-GAN + Transformer (3D GAN variant of [21])	0.424	0.301
VQ-GAN + Transformer + CT conditioning (ours)	0.468	0.344
VQ-GAN + Transformer + CT conditioning + KDE (ours)	0.505	**0.501**
VQ-GAN + Transformer + CT conditioning + KDE + 40% Thresholding (ours)	**0.575**	0.458

## References

[1] Almuhaideb A, Papathanasiou N, Bomanji J 18f-fdg pet/ct imaging in oncology. Annals of Saudi medicine.

[2] Baur C, Denner S, Wiestler B, Albarqouni S, Navab N (2020). Autoencoders for unsupervised anomaly segmentation in brain mr images: A comparative study.

[3] Burgos N, Cardoso MJ, Samper-González J, Habert MO, Durrleman S, Ourselin S, Colliot O, Initiative, A.D.N., Initiative, F.L.D.N (2021). Anomaly detection for the individual analysis of brain pet images. Journal of medical imaging (Bellingham, Wash).

[4] Chen CF, Fan Q, Panda R (2021). Crossvit: Cross-attention multi-scale vision transformer for image classification.

[5] Chen M, Radford A, Wu J, Heewoo J, Dhariwal P (2020). Generative pretraining from pixels.

[6] Child R, Gray S, Radford A, Sutskever I (2019). Generating long sequences with sparse transformers.

[7] Choromanski K, Likhosherstov V, Dohan D, Song X, Gane A, Sarlos T, Hawkins P, Davis J, Mohiuddin A, Kaiser L, Belanger D (2020). Rethinking attention with performers.

[8] Dhariwal P, Jun H, Payne C, Kim JW, Radford A, Sutskever I (2020). Jukebox: A generative model for music.

[9] Dumoulin V, Belghazi I, Poole B, Mastropietro O, Lamb A, Arjovsky M, Courville A (2016). Adversarially learned inference.

[10] Esser P, Rombach R, Ommer B (2020). Taming transformers for high-resolution image synthesis.

[11] Gheini M, Ren X, May J (2021). Cross-attention is all you need: Adapting pretrained transformers for machine translation.

[12] Jun H, Child R, Chen M, Schulman J Distribution augmentation for generative modeling.

[13] Kim HS, Lee KS, Ohno Y, van Beek EJR, Biederer J (2015). Pet/ct versus mri for diagnosis, staging, and follow-up of lung cancer. Journal of magnetic resonance imaging : JMRI.

[14] Liu B, Gao S, Li S (2017). A comprehensive comparison of ct, mri, positron emission tomography or positron emission tomography/ct, and diffusion weighted imaging-mri for detecting the lymph nodes metastases in patients with cervical cancer: A meta-analysis based on 67 studies. Gynecologic and Obstetric Investigation.

[15] Marimont SN, Tarroni G (2020). Anomaly detection through latent space restoration using vector-quantized variational autoencoders.

[16] Mohla S, Pande S, Banerjee B, Chaudhuri S (2020). Fusatnet: Dual attention based spectrospatial multimodal fusion network for hyperspectral and lidar classification.

[17] Newman-Toker DE, Wang Z, Zhu Y, Nassery N, Tehrani ASS, Schaffer AC, Yu-Moe CW, Clemens GD, Fanai M, Siegal D (2021). Rate of diagnostic errors and serious misdiagnosis-related harms for major vascular events, infections, and cancers: toward a national incidence estimate using the “big three”. Diagnosis (Berlin, Germany).

[18] van den Oord A, Vinyals O, Kavukcuoglu K (2017). Neural discrete representation learning.

[19] Parzen E (1962). On estimation of a probability density function and mode. The Annals of Mathematical Statistics.

[20] Perani D, Daniela P, Schillaci O, Orazio S, Padovani A, Alessandro P, Nobili FM, Mariano NF, Iaccarino L, Leonardo I, Rosa PAD (2014). A survey of fdg- and amyloid-pet imaging in dementia and grade analysis. BioMed research international.

[21] Pinaya WHL, Tudosiu PD, Gray R, Rees G, Nachev P, Ourselin S, Cardoso MJ (2021). Unsupervised brain anomaly detection and segmentation with transformers.

[22] Radford A, Narasimhan K (2018). Improving language understanding by generative pre-training.

[23] Takaki S, Nakashika T, Wang X, Yamagishi J (2018). Stft spectral loss for training a neural speech waveform model.

[24] Tay Y, Dehghani M, Abnar S, Shen Y, Bahri D, Pham P, Rao J, Yang L, Ruder S, Metzler D (2020). Long range arena: A benchmark for efficient transformers.

[25] Vaswani A, Shazeer N, Parmar N, Uszkoreit J, Jones L, Gomez AN, Kaiser L, Polosukhin I (2017). Attention is all you need.

[26] Yan W, Zhang Y, Abbeel P, Srinivas A (2021). Videogpt: Video generation using vq-vae and transformers.

[27] Zhang R, Isola P, Efros AA, Shechtman E, Wang O (2018). The unreasonable effectiveness of deep features as a perceptual metric.

