# Supplementary material for: Cross Attention Transformers for Multi-modal Unsupervised Whole-Body PET Anomaly Detection
Source: Deep Gener Model (2022). Author manuscript; Available in PMC 2024 Sep 20. (PMC7616582; doi:10.1007/978-3-031-18576-2_2)
Supplement: Appendix [file EMS198179-supplement-Appendix.pdf]

A Dataset

To assess the performance of the anomaly detection methods proposed, we utilised 12 paired whole-body PET/CT images with varying cancers across samples. From the original private dataset of 83 whole-body PET/CT samples, 60 were used for training, 11 were used for validation to tune model hyperparameters and anomaly detection parameters, including the transformer latent code resampling threshold and minimum bandwidth of the KDE approach. The remaining 12 were set aside to be used for testing only. All scans had a field of view from the neck down to their upper thigh region and were registered to a group space using a rigid alignment. The voxel dimensions of each scan were $216 \times 168 \times 208$.

B VQ-GAN Implementation

The VQ-VAE model uses a discrete codebook of learned representations so that an image $x \in \mathbb{R}^{H \times W \times D}$ can be represented by the codebook entries $\hat{z} \in \mathbb{R}^{h \times w \times d \times n_z}$ where n_z is the size of each latent vector. After the encoder network projects the image x to its latent representation, each feature vector is quantized by a nearest neighbour look-up. The posterior distribution can then be given as a categorical one defined as:

$$q(\hat{z}_{ijl} = k|x) = \begin{cases} 1 \; for \; k = argmin_c \left\| \hat{z}_{ijl}(x) - e_c \right\|_2 \\ 0 \qquad\qquad otherwise \end{cases} \tag{3}$$

Where $\hat{z}_{ijl}(x)$ is the output from the encoder, and e_c is a codebook vector in the shared embedding space. The total loss for the VQ-VAE is then given as:

$$L_{VQVAE} = \|(\mathbf{x} - \hat{\mathbf{x}})\|_2^2 + \||STFT(\mathbf{x})| - |STFT(\hat{\mathbf{x}})|\|_2^2 + \beta \, \|z_e(\mathbf{x}) - sg[\mathbf{e}]\|_2^2 + \|sg[z_e(\mathbf{x})] - \mathbf{e}\|_2^2 \tag{4}$$

where sg stands for a stopgradient operator to stop gradients from flowing back into their argument. The loss function for the VQ-VAE makes use of a spectral loss [8] that is, it includes a component based off of the magnitude of the Fourier transform of the original and reconstructed image. From equation 4 the first term is the pixel loss, the second term is the spectral loss between the original and reconstruction where SFTF stands for the short time Fourier transform. The third term is the commitment cost used to ensure the encoder commits to the codebook. The final term is to move the codebook embedding vectors towards the output from the encoder. For this term, we replace this and use the exponential moving average updates for the codebook [18]. During training, a β of 0.25 was used.

When implementing the VQ-GAN network however due to instabilities associated with adversarial networks, the loss function is further amended to include a perceptual loss [23] that helps to preserve spatial consistency, making use of the lpips library [27]. This loss is randomly applied to 50% of slices across each plane.

The architecture used for the VQ-GAN model makes use of an encoder consisting of three strided convolutional layers with stride 2 and kernel size 4. Each convolutional layer is then followed by a ReLU activation and 3 residual blocks (consisting of a 3x3x3 conv, ReLU, 1x1x1 conv, ReLU). The decoder similarly has 3 residual blocks, each followed by a transposed convolutional layer with stride 2 and kernel size 4. Finally, before the last transposed convolutional layer a Dropout layer with a probability of 0.05 is added. Additionally the codebook for the PET VQ-GAN had 64 atomic elements, each of length 256, whilst the CT VQ-GAN had a total of 256 atomic elements each of length 256. To train the VQ-GAN network, we used an ADAM optimiser with a learning rate of 1e-4, an exponential learning rate decay with a gamma of 0.9999. Additionally the discriminator network had a learning rate of 5e-4. Training data was augmented using elastic deformations, Gaussian noise, intensity shifts and contrast adjustments. The model was trained over 2000 epochs with a batch-size of 3.

C Transformer Implementation

The transformer model, relies on attention mechanisms to learn sequential data. The self-attention mechanism is best described as a mapping of intermediate representations of three position-wise linear layers onto three representations denoted by the Value (V), key (K) and query (Q), [25]. With d_k denoting the key dimension of the output, the attention mechanism is calculated as:

$$Attn(Q, K, V) = softmax\left(\frac{QK^T}{\sqrt{d_k}}\right)V \tag{5}$$

The multi-head attention aspect of this transformer network is then several attention layers run in parallel with their outputs concatenated and fed through a linear layer. To add cross attention to this architecture, we add a cross attention layer after each self-attention layer in the transformer architecture. Still using the same attention mechanism the cross attention calculation is then given as:

$$Attn(Q_s, K_c, V_c) = softmax\left(\frac{Q_s K_c^T}{\sqrt{d_k}}\right)V_c \tag{6}$$

Where Q_s is the output from the prior self-attention layer and K_c and V_c are the Key and Query values derived from the embedded conditioning CT sequence.

The performer used corresponds to a decoder transformer architecture with 14 layers (each layer consisting of a self-attention and cross-attention mechanism), each with 8 heads, and an embedding size of 256.

To train the performer network, we used an ADAM optimiser with a learning rate of 1e-3, an exponential learning rate decay with a gamma of 0.9999. The loss function used for training was cross-entropy given the discrete nature of the latent sequence codes. Additionally training data was augmented a total of 4 times using elastic deformations, Gaussian noise, intensity shifts and contrast adjustments to render 240 augmented training samples. The model was trained over 200 epochs with a batch-size of 1.
